# Supplementary material for: On-Chip Optical Beam Manipulation with an Electrically Tunable Lithium-Niobate-on-Insulator Metasurface
Source: Micromachines (Basel). 2022 Mar 19;13(3):472. doi: 10.3390/mi13030472 (PMC8950189; doi:10.3390/mi13030472)
Supplement: Supplementary file 1 [file micromachines-13-00472-s001.zip › micromachines-1626941-supplementary.pdf]

micromachines

Supplementary Information

On-Chip Optical Beam Manipulation with an Electrically Tunable Lithium-Niobate-on-Insulator Metasurface

Linyuan Dou [1,†], Lingyun Xie [1,2,3,*,†], Zeyong Wei [1,2,3,*], Zhanshan Wang [1,2,3] and Xinbin Cheng [1,2,3]

1 Institute of Precision Optical Engineering, School of Physics Science and Engineering, Tongji University, Shanghai 200092, China; 1852053@tongji.edu.cn (L.D.); wangzs@tongji.edu.cn (Z.W.); chengxb@tongji.edu.cn (X.C.)
2 MOE Key Laboratory of Advanced Micro-Structured Materials, Shanghai 200092, China
3 Shanghai Frontiers Science Research Base of Digital Optics, Tongji University, Shanghai 200092, China
* Correspondence: 0107xielingyun@tongji.edu.cn (L.X.); weizeyong@tongji.edu.cn (Z.W.)
† These authors contributed equally to this work are considered co-first authors.

1. Distribution of refractive index variation of the proposed device

In the focusing function, no voltage is applied to the unit in the middle of the device. Because of the symmetry of the phase, the voltages applied to the units in the symmetrical position is equal. Therefore, there are 9 units in which different voltages are applied. Below 4 tables are used to present the distribution of refractive index variation among these 9 units required for the focusing function in Figure 3.

Table S1. Distribution of refractive index variation among the units when $\Delta n_{\mathrm{omax}} = -0.05$. [1]

Unit number	y coordinate (μm) [2]	Δn_o [3]	Δn_e [4]
1	0.7	−0.0006	−0.0019
2	1.4	−0.0025	−0.0077
3	2.1	−0.0056	−0.0173
4	2.8	−0.0099	−0.0307
5	3.5	−0.0154	−0.0480
6	4.2	−0.0222	−0.0691
7	4.9	−0.0302	−0.0940
8	5.6	−0.0395	−0.1228
9	6.3	−0.0500 [1]	−0.1554

1 Δn_{omax} refers to the maximum ordinary refractive index variation in all units.
2 The y coordinate value of the center of the units.
3 Ordinary refractive index variation. Variation is calculated based on that $n_o = 2.211$ (@1550nm) with no voltage applied.
4 Extraordinary refractive index variation. Variation is calculated based on that $n_e = 2.138$ (@1550nm) with no voltage applied.

Table S2. Distribution of refractive index variation among the units when $\Delta n_{omax} = -0.10$.

Unit number	y coordinate (μm)	Δn_o	Δn_e
1	0.7	−0.0012	−0.0038
2	1.4	−0.0049	−0.0153
3	2.1	−0.0111	−0.0345
4	2.8	−0.0198	−0.0614
5	3.5	−0.0309	−0.0959
6	4.2	−0.0444	−0.1381
7	4.9	−0.0605	−0.1880
8	5.6	−0.0790	−0.2455
9	6.3	−0.1000	−0.3108

Table S3. Distribution of refractive index variation among the units when $\Delta n_{omax} = -0.15$.

Unit number	y coordinate (μm)	Δn_o	Δn_e
1	0.7	−0.0019	−0.0058
2	1.4	−0.0074	−0.0230
3	2.1	−0.0167	−0.0518
4	2.8	−0.0296	−0.0921
5	3.5	−0.0463	−0.1439
6	4.2	−0.0667	−0.2072
7	4.9	−0.0907	−0.2820
8	5.6	−0.1185	−0.3683
9	6.3	−0.1500	−0.4662

Table S4. Distribution of refractive index variation among the units when $\Delta n_{omax} = -0.20$.

Unit number	y coordinate (μm)	Δn_o	Δn_e
1	0.7	−0.0025	−0.0077
2	1.4	−0.0099	−0.0307
3	2.1	−0.0222	−0.0691
4	2.8	−0.0395	−0.1228
5	3.5	−0.0617	−0.1918
6	4.2	−0.0889	−0.2762
7	4.9	−0.1210	−0.3760
8	5.6	−0.1580	−0.4911
9	6.3	−0.2000	−0.6215

In the deflecting function, voltages are applied to only 9 units in the middle of the device. Below 2 tables are used to present the distribution of refractive index variation among these 9 units required for the deflecting function in Figure 5.

Table S5. Distribution of refractive index variation among the units when the deflection of 30° is realized.

Unit number[1]	y coordinate (μm)	Δn_o	Δn_e
1	2.8	−0.0053	−0.0164
2	2.1	−0.0106	−0.0328
3	1.4	−0.0158	−0.0492
4	0.7	−0.0211	−0.0656
5	0	−0.0264	−0.0820
6	−0.7	−0.0317	−0.0984
7	−1.4	−0.0369	−0.1148
8	−2.1	−0.0422	−0.1312
9	−2.8	−0.0475	−0.1476

[1] These 9 units are in the middle of the total device.

Table S6. Distribution of refractive index variation among the units when the deflection of 45° is realized.

Unit number[1]	y coordinate (μm)	Δn_o	Δn_e
1	2.8	−0.0108	−0.0335
2	2.1	−0.0216	−0.0670
3	1.4	−0.0323	−0.1005
4	0.7	−0.0431	−0.1340
5	0	−0.0539	−0.1675
6	−0.7	−0.0647	−0.2010
7	−1.4	−0.0754	−0.2345
8	−2.1	−0.0862	−0.2680
9	−2.8	−0.0970	−0.3014
